# Host Matters: Medicinal Leech Digestive-Tract Symbionts and Their Pathogenic Potential

**DOI:** 10.3389/fmicb.2016.01569

**Published:** 2016-10-13

**Authors:** Jeremiah N. Marden, Emily A. McClure, Lidia Beka, Joerg Graf

**Affiliations:** ^1^Department of Molecular and Cell Biology, University of Connecticut, StorrsCT, USA; ^2^Institute for Systems Genomics, University of Connecticut, StorrsCT, USA

**Keywords:** *Aeromonas*, *Hirudo*, digestive-tract symbiosis, bacteroidetes, leech therapy, mucinivorans, beneficial bacteria

## Abstract

Digestive-tract microbiota exert tremendous influence over host health. Host-symbiont model systems are studied to investigate how symbioses are initiated and maintained, as well as to identify host processes affected by resident microbiota. The medicinal leech, *Hirudo verbana*, is an excellent model to address such questions owing to a microbiome that is consistently dominated by two species, *Aeromonas veronii* and *Mucinivorans hirudinis*, both of which are cultivable and have sequenced genomes. This review outlines current knowledge about the dynamics of the *H. verbana* microbiome. We discuss in depth the factors required for *A. veronii* colonization and proliferation in the leech crop and summarize the current understanding of interactions between *A. veronii* and its annelid host. Lastly, we discuss leech usage in modern medicine and highlight how leech-therapy associated infections, often attributable to *Aeromonas* spp., are of growing clinical concern due in part to an increased prevalence of fluoroquinolone resistant strains.

## Introduction

The human digestive-tract microbiota is implicated in affecting circadian rhythms, cancer, obesity, pharmacokinetics, and even mental health (Spanogiannopoulos et al., 2016). Investigating naturally occurring, simple, tractable model symbioses allows the identification of molecular mechanisms that through comparative studies can be generalized (Ruby, 2008) and applied to humans. One such model is the medicinal leech. The leech has a number of aspects making it suitable for molecular studies, including the presence of a simple microbial community whose dominant members can be cultured (Graf et al., 2006; Nelson and Graf, 2012).

Leeches are fascinating animals whose ability to consume blood from vertebrate hosts has been used to treat a wide range of diseases for millennia (Graf, 2000; Müller, 2000). Since the 1980’s, medicinal leech use in Western Europe and the United States has made a resurgence, especially in the treatment of vascular congestion after reconstructive surgery (de Chalain, 1996; Whitaker et al., 2004a, 2011). However, clinical use of leeches in many cases leads to wound infections that are presumably caused by digestive-tract symbiont(s) (Whitlock et al., 1983; Lineaweaver et al., 1992; Bauters et al., 2007; Whitaker et al., 2011). Historically, *Pseudomonas hirudinis* (now reclassified as *Aeromonas hydrophila*) was reported to be the only culturable bacterium from the crop (Büsing, 1951). More recent studies reveal a moderately complex community dominated by *Aeromonas veronii* and *Mucinivorans hirudinis* (Graf, 1999; Worthen et al., 2006; Maltz et al., 2014).

Studies of microbe–host interactions are aided by an ability to culture the symbionts, manipulate associations, perform genetics on the partners, and have access to the partners’ genome sequences (Ruby, 2008; Graf, 2016). *H. verbana* and its symbionts meet many of these criteria (Nelson and Graf, 2012): dominant symbionts are culturable (Graf, 1999; Bomar et al., 2011); genetic tools are available for *A. veronii* (Rio et al., 2007; Silver et al., 2007b; Maltz et al., 2015); the microbe–host association can be manipulated through antibiotic treatment and feeding of microbial species of interest (Graf, 1999; Mumcuoglu et al., 2010); genomes, metagenomes, and metatranscriptomes for the symbionts are available (Bomar et al., 2011, 2013; Bomar and Graf, 2012; Maltz et al., 2014; Nelson et al., 2015a); and an EST library for the host is also available (Macagno et al., 2010). The successful application of these tools has made the leech an amenable and powerful model for studying digestive-tract symbioses. In this review we outline current knowledge regarding microbial symbioses within the leech digestive tract, summarize known colonization factors of the dominant symbiont, *A. veronii*, and discuss current practices and precautions associated with medicinal leech treatment.

## The Medicinal Leech

The most commonly available medicinal leech in the United States is *H. verbana*, although it is often mislabeled as *H. medicinalis* by medical suppliers (Siddall et al., 2007a). This confusion stems from a recent clarification of *Hirudo* taxonomy and the challenge of differentiating species solely based on pigmentation patterns. *Hirudo* species are native to Africa, Asia and Europe: *H. orientalis* (Transcaucasia and Iran), *H. nipponia* (East Asia), *H. troctina* (North Africa), *H. verbana* (Southeastern Europe and Turkey), and *H. medicinalis* (continental Europe and Britain) (Sawyer, 1986; Siddall et al., 2007a; Trontelj and Utevsky, 2012). In order to accurately identify a given species, DNA barcoding using the cytochrome C oxidase subunit 1 gene is recommended (Siddall et al., 2007a). Although leech species differ in salivary protein (Baskova et al., 2008; Siddall et al., 2011) and gut microbiota composition (Graf, 1999; Siddall et al., 2007b; Laufer et al., 2008; Whitaker et al., 2014), it remains unknown whether or not the efficacy of leech therapy is dependent on the leech species used.

**FIGURE 1:**
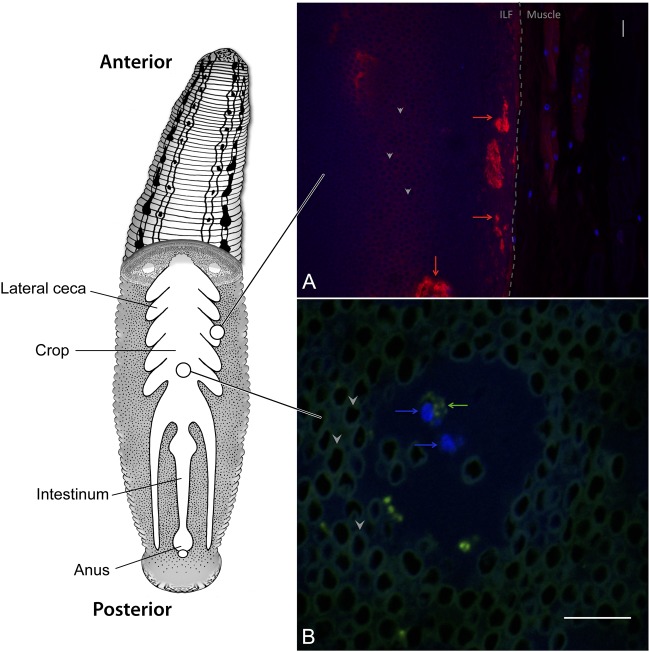
***Hirudo verbana* Digestive Tract.** Schematic of the leech digestive tract (modified from Nelson and Graf, 2012 and Maltz et al., 2014). The ingested blood meal is stored in the crop where it forms a highly viscous intraluminal fluid (ILF) consisting of densely packed erythrocytes (dark circles surrounded by autofluorescence, examples indicated with arrow heads in insets). Fluorescence *in situ* hybridization micrographs of the leech crop describe **(A)** thick layers of mucus (red arrows) near the crop epithelium (dashed line) that develop after feeding and **(B)** circulating hemocytes (blue arrows) within the ILF that contain bacterial cells (green arrows). DAPI (blue), sWGA (red), and EUB338 (green). Scale bars = 10 μm.

The leech digestive tract is comprised of three major regions, the pharynx, crop, and intestinum, with each region performing distinct functions (**Figure 1**) (Sawyer, 1986). The pharynx is a muscular region located immediately downstream of the jaws and adjacent to the salivary glands. The largest compartment of the digestive tract is the crop, where ingested blood meals are stored and from which water and osmolytes are removed (Wenning et al., 1980). The removal of water concentrates the blood meal and forms a highly viscous intraluminal fluid (ILF). Pairs of bladders flank each cecum in the crop, facilitate the removal of water, and are themselves colonized by a distinct microbial community (Wenning and Cahill, 1989; Kikuchi et al., 2009). Digestion occurs over several weeks and is thought to occur mostly in the intestinum. The leech’s anatomy allows it to ingest a sizeable blood meal upon encountering its prey, accommodating up to five times its body weight of blood in a single meal (Wenning et al., 1980). Ingested erythrocytes are stored in the crop, remaining visually intact over prolonged time periods despite the presence of bacteria capable of β-hemolysis (**Figure 1**). Due to effective storage and slow digestion, the leech can go for 6 months between feedings (Sawyer, 1986).

## The Leech Crop Microbiota

To date, the composition of the gut microbiota from *H. verbana, H. medicinalis*, and *H. orientalis* have been studied. In each host species, the microbial community is dominated by *Aeromonas* and *Bacteroidetes* spp. (Worthen et al., 2006; Siddall et al., 2007b, 2011; Laufer et al., 2008; Whitaker et al., 2014). In *H. verbana*, the predominant *Bacteroidetes* species was initially termed *Rikenella-*like and was recently renamed as *Mucinivorans hirudinis*, a member of the *Rickenellaceae* (Worthen et al., 2006; Nelson et al., 2015b). Phylogenetic analysis of 16S rRNA gene sequences of *Bacteroidetes* isolates from hirudiniform leeches suggests close evolutionary relationships between the leech species and their *Bacteroidetes* symbionts (Siddall et al., 2011).

*M. hirudinis* is a member of the family *Rikenellaceae* along with *Alistipes, Anaerocella*, and *Rikenella* (Graf, 2014). These bacteria are capable of anaerobic metabolism and utilize carbohydrates as carbon and energy sources. *M. hirudinis* can ferment glucose, lactose, mannose, and melibiose and the metabolic endproducts include alcohols, acetic acid, proprionic acid, and succinic acid (Nelson et al., 2015b). In addition, *M. hirudinis* can metabolize mucus (Bomar et al., 2011), which likely provides it with an advantage in colonizing digestive tracts.

In contrast to highly specialized symbiotic bacteria that must live in close association with their host, *Aeromonas* spp. can succeed in a wide range of habitats (Martin-Carnahan and Joseph, 2005). *A. veronii* is capable of proliferating as a symbiont in digestive tracts of leeches and zebrafish (Bates et al., 2007; Roeselers et al., 2011; Nelson and Graf, 2012), subsisting as free-living cells within aquatic environments, and causing diseases in fish and mammals (Janda and Abbott, 2010; Beaz-Hidalgo and Figueras, 2013; Hossain et al., 2014). This ability to associate with different hosts provides an excellent opportunity to compare mechanistic aspects of bacterial virulence and mutualistic associations (Hentschel et al., 2000; Silver et al., 2007a).

The origin of bacterial symbionts in the leech crop has been evaluated using diagnostic PCR-based to determine whether symbionts are transmitted vertically from parent leech to offspring. The results suggested that *Aeromonas* is already associated with leech embryos inside the cocoon and that *Mucinivorans* reached detectable levels after hatching (Rio et al., 2009). Two additional studies using GFP-labeled *A. veronii* indicated that bacteria present on the mucosal castings could enter the digestive tract of antibiotic-cured adult leeches (Ott et al., 2014, 2016). These findings suggest that multiple modes of transmission of *Aeromonas* to juveniles may exist.

Because *H. verbana* has been studied in the greatest detail, this review will focus specifically on the symbionts of this host. In addition to the two dominant symbionts, *A. veronii* and *M. hirudinis*, other reported genera include: *Morganella, Clostridium, Erysipelothrix, Desulfovibrio*, and *Fusobacterium* (Worthen et al., 2006; Maltz et al., 2014). Of these bacteria, *Aeromonas, Mucinivorans, Morganella, Clostridium*, and *Desulfovibrio* were also found in *H. orientalis* (Whitaker et al., 2014). The prevalence of these bacteria in hirudiniform leeches immediately after being captured in the wild needs to be evaluated to gather a better understanding of naturally occurring microbial diversity.

## Nutrient Acquisition and Metabolism

Bacteria capable of occupying multiple habitats must generally be able to acquire and metabolize diverse nutrients. One of the most basic colonization barriers to symbionts and non-symbionts alike is the need to compete for and utilize available food sources (Graf, 2016). One approach to identify these resources is to screen for mutants with a reduced ability to colonize. Using signature-tagged mutagenesis (STM), one can screen multiple mutants in one animal. A mixture of mutants is introduced and they compete for nutrients and other resources inside the host (Hensel et al., 1995). For verifying the colonization capability of mutants, an individual mutant and a competitor strain are fed to the leech and forced to compete against each other and the native microbiota. The output and input ratios are used to calculate a competitive index (CI) where a ratio of less than one indicates a colonization defect of the mutant. The metabolic capacity of the organism and the resulting ability to outcompete other organisms inside the host niche are an important determinant of successful host colonization.

### Mucus

The digestive tracts of many animals are lined with mucus, which protects the underlying epithelium and serves as a nutrient source for some digestive-tract symbionts, (e.g., *Bacteroides thetaiotaomicron* and *Akkermansia muciniphila*) (Tailford et al., 2015). The epithelium of the leech crop is also covered with mucus, which increases in thickness after feeding (Bomar et al., 2011) (**Figure 1A**). Fluorescence *in situ* hybridization (FISH) imaging shows that *M. hirudinis* associates with mucus lining the leech crop epithelial wall and the abundance of surface-associated cells increases after the leech consumes a blood meal (Kikuchi and Graf, 2007). A metatranscriptomic analysis of the *H. verbana* ILF revealed that *M. hirudinis* expresses genes involved in mucin and glycan utilization at a level exceeding that of ribosomal protein coding genes. This information was exploited to design media containing mucin as the sole carbon source in order to propagate this bacterium. This optimized media was successfully used to culture the bacterium, which had previously proven recalcitrant to cultivation (Bomar et al., 2011). It is hypothesized that acetate, a fermentation product released by *M. hirudinis* is used as an energy source by *A. veronii* (Bomar et al., 2011). It is unknown whether increased mucus expression is driven by the utilization of mucin by *M. hirudinis*, as was shown for *B. thetaiotaomicron* inducing the biosynthesis of fucose in the mouse gut (Hooper et al., 1999), or if the leech simply produces mucus after feeding to protect its epithelium.

### Proteins

Blood is a rich nutrient source high in proteins, particularly albumin. Two lines of evidence suggest that *A. veronii* catabolizes the highly concentrated proteins in the leech crop. Firstly, a STM screen identified a mutant with a significantly lower CI in the crop (**Table 1**). This mutant had a disruption of *tdcC*, a conserved, anaerobically induced, threonine/serine transporter (Silver et al., 2007b), suggesting an increased competition for these amino acids in the leech crop. The second line of evidence for proteins being utilized as a nutrient in the leech crop is based on an analysis of metatranscriptome data. In this study high expression levels of genes associated with arginine catabolism, *arcABCD*, were detected during colonization (Bomar et al., 2011). These genes encode proteins whose products have roles in catabolism of arginine, a poor energy source, via the arginine deiminase pathway (Bomar et al., 2011). Collectively these data suggest that the proteins present in blood are an important nutrient for the digestive–tract symbionts.

**Table 1:** *Aeromonas veronii* colonization mutants.

Functional category	Strain(s)	Predicted function of disrupted or identified locus	Competition Defect	Reference
Complement resistance	> 30 isolates	Multiple loci	+, ++, +++	Silver et al., 2007b
Oxidative stress response	JG186	(KatA)	–	Rio et al., 2007
Surface modification	JG535	Glycosyltransferase, type 1 capsular polysaccharide synthesis	+++	Silver et al., 2007b
	JG730 JG736	Murein lipoprotein (*lpp*)	+++	Silver et al., 2007b
	JG735	3-deoxy-D-manno-octulosonic-acid transferase	+++	Silver et al., 2007b
	JG738	Polysaccharide synthesis protein/Glycosyltransferase (WbbB)	++	Silver et al., 2007b
Regulatory	JG547	Ribosomal operon	+	Silver et al., 2007b
	JG697	GTPase (YchF)	++	Silver et al., 2007b
	JG741	RNase II	+	Silver et al., 2007b
Nutrition	JG537	Phosphate ABC transporter (PstC)	++	Silver et al., 2007b
	JG698	ZIP family metal transporter (ZupT)	+	Silver et al., 2007b
	JG750	Threonine/serine transporter (TdcC)	++	Silver et al., 2007b
Host interaction	JG752	T3SS apparatus (AscU)	++	Silver et al., 2007a
	JG573	T6SS effector, Rearrangement hotspot protein (Rhs)	+++	Silver et al., 2007b
	HE-1095	T2SS apparatus (ExeM)	+	Maltz and Graf, 2011
Unknown	JG521 JG523 JG538	Hypothetical proteins	+	Silver et al., 2007b
	JG532	MBL-fold metallo-hydrolase domain containing protein	+	Silver et al., 2007b
	JG533	KAP family P-loop NTPase protein	+	Silver et al., 2007b
	JG751	Intergenic region; upstream of predicted GTPase	+	Silver et al., 2007b
	JG753	Intergenic region; upstream of hypothetical protein	+	Silver et al., 2007b


*Competition defect of *Aeromonas* isolates in the leech crop relative to the parent strain is indicated as follows: –, no colonization deficiency; +, 2–10 fold deficiency; ++, 10–100 fold deficiency; +++, >100 fold deficiency.*

### Erythrocytes

A major source of nutrients in the blood meal is erythrocytes. Interestingly the erythrocytes in the leech crop are maintained intact for months after feeding despite the presence of bacteria capable of lysing blood cells (Sawyer, 1986; Lent et al., 1988; Maltz and Graf, 2011). Insight into maintenance of erythrocyte integrity was provided by a transposon mutant screen, which generated a single *Aeromonas* mutant unable to perform β-hemolysis (Maltz and Graf, 2011). The mutation mapped to *exeM*, a type 2 secretion system (T2SS) component, and resulted in a significantly reduced ability to colonize the leech crop (**Table 1**). The colonization defect was alleviated by feeding leeches partially lysed blood. A likely explanation of this phenotypic complementation is that the loss of β-hemolysis was responsible for the colonization defect due to the lack of liberated protein, lipids, and heme from the lysed erythrocytes.

### Lipids

The lysis of ingested erythrocytes likely provides an ample source of lipids. *A. veronii* colonizing the leech crop show elevated expression of malate synthase and isocitrate lyase. These enzymes are critical in the glyoxylate shunt, responsible for lipid metabolism (Bomar et al., 2011), and their expression suggests that in the leech crop *A. veronii* utilizes short-chained fatty acids (SCFA), such as acetate, and/or β-oxidation of fatty acids (Bomar et al., 2011). These nutrients are likely by-products of glycan fermentation by *M. hirudinis* or obtained from erythrocyte membranes, respectively.

### B-vitamins

Although blood is a high-energy nutrient source it is notably deficient in B-vitamins (Lehane, 1991). Biosynthesis of these vitamins by symbiotic bacteria is thought to supplement the dietary requirements of exclusively sanguivorous organisms. In order to foster this association, some leeches possess a specialized organ, a mycetome, which houses highly adapted intracellular bacteria (Kikuchi and Fukatsu, 2002; Siddall et al., 2004). An example of such a highly adapted symbiont is *Providencia siddallii*, an endosymbiont of *Haementeria officinalis*, with a reduced genome lacking canonical synthesis pathways for all essential amino acids while maintaining those which produce most cofactors and B-vitamins (Manzano-Marin et al., 2015). The capacity for biosynthesis of B-vitamins is observed in the endosymbionts of many obligate blood-feeders (Manzano-Marin et al., 2015) and is believed to be possessed by the digestive-tract symbionts in *H. verbana*, which lacks a mycetome (Maltz et al., 2014; Nelson et al., 2015a). For instance, the genome of *M. hirudinis* suggests a capability of producing cobinamide, a precursor of the vitamin B12 coenzyme (Nelson et al., 2015a). Without its microbial symbionts, subsisting exclusively on a blood diet would probably be impossible for *H. verbana*.

### Heme/Iron

As blood contains very high levels of heme, organisms that feed on blood must have mechanisms to counter heme toxicity (Graça-Souza et al., 2006). One aspect of this damage is Fenton reaction-mediated oxidative stress due to the release of iron from the heme moieties. In some hemipterans, haemoxisomes in epithelial cells lining the gut protect the animal by sequestering heme (Silva et al., 2006). In the North American leech, *Macrobdella decora*, this is accomplished with His-rich proteins (Min et al., 2010), though whether *H. verbana* possesses homologs has yet to be ascertained. Sequestration of iron by transferrin in plasma and by hemoglobin in erythrocytes not only prevents oxidative damage to the host, but also restricts an essential nutrient for bacterial growth (Schaible and Kaufmann, 2004). Bacteria can acquire protein-bound iron either by producing high affinity siderophores or proteins that bind iron-containing host proteins and mediate their uptake (Byers et al., 1991).

The ability to lyse erythrocytes in order to acquire iron from the released heme is critical for allowing rapid proliferation of *Aeromonas* during leech colonization (Maltz and Graf, 2011; Maltz et al., 2015). Disruption of *hgpB*, an outer membrane heme receptor, or an associated transcriptional activator, *hgpR*, prevents *Aeromonas* from obtaining heme-associated iron and colonizing the leech digestive tract (Maltz et al., 2015). In contrast, disrupting *viuB* (vibrobactin utilization protein), which mediates transport of siderophore acquired iron, did not affect colonization of the crop (Maltz et al., 2015). These data suggest that attaining iron from heme is crucial for the ability of *A. veronii* to colonize the leech. Interestingly, heme utilization genes are widely distributed among *Aeromonas* species and isolates obtained from different sources, suggesting that they may play an additional role outside of symbiosis (Maltz et al., 2015).

### Other Nutrients

In addition to carbon and energy sources, sufficient amounts of minor nutrients and metals can also be important for rapid growth inside the host. The STM screen identified two additional strains with nutrient-acquistion related genes that were disrupted (Silver et al., 2007b). JG537 is mutated in a *pstC* homolog, encoding a phosphate specific ABC transporter permease (**Table 1**). While the observed competition defect may be attributable to phosphate starvation, an alternative explanation of decreased membrane stability cannot be ruled out since both phenotypes are linked to disruption of this apparatus (Rao and Torriani, 1990; Daigle et al., 1995; Aguena et al., 2002; Lamarche et al., 2005). The other mutant, JG698, has a transposon within a gene encoding a ZupT ZIP protein family permease (**Table 1**). ZupT from *Escherichia coli* has been shown to have broad specificity for diverse cations, with an overall preference for zinc (Grass et al., 2002, 2005; Taudte and Grass, 2010). The reduced CI in both the leech and blood control of this mutant indicate that this transporter has a more general growth defect rather than being leech-specific (Silver et al., 2007b). However, if the leech locally restricts cation availability in the crop in response to colonization, the ZupT homolog may have a role in overcoming that response.

## Colonization Dynamics

The growth of symbionts *in vivo* is affected by the ability to utilize nutrients, evade the host immune response, and compete with resident microbes. In the leech, the infrequent consumption of blood meals leads to particular bacterial growth dynamics inside the crop. After feeding, *A. veronii* and *M. hirudinis* rapidly proliferate for ∼3 days before entering a quiescent state marked by an increase in expression of stress-response related genes and a gradual decrease in population size of *A. veronii* while *M. hirudinis* continues to increase more gradually, peaking at ∼7 days (Kikuchi and Graf, 2007; Bomar and Graf, 2012). After the initial rapid proliferation, the populations of both symbionts gradually decrease in abundance and return to levels found in the starved state. For *A. veronii* this decline occurs within 14 days while the abundance of *M. hirudinis* drops at a slower rate (Kikuchi and Graf, 2007).

A major hallmark of the *A. veronii* transition into a quiescent state is the upregulation of the ncRNAs CsrB and CsrC (carbon starvation response). Both ncRNAs negatively regulate the mRNA binding translational regulator CsrA. CsrA/B/C homologs are found in diverse bacteria and control numerous processes such as metabolism, biofilm formation, and virulence factor production (Vakulskas et al., 2015). CsrA binds and regulates translation of target mRNA transcripts, but is antagonized by CsrB/C, which contain high affinity CsrA binding sites. A comparative *in vitro* and *in vivo* RNAseq transcriptome analysis revealed dramatically higher levels of CsrB and CsrC (>50 fold) inside the leech crop than when cultured to stationary phase in a rich medium (Bomar and Graf, 2012). This observation illustrates that the global control over mRNA and protein production is a vital aspect of the processes by which *Aeromonas* adapts to growth inside the leech crop.

## Immunity

In persistent symbiotic relationships between bacteria and animals, the symbionts are in a stalemate or a détente (McFall-Ngai, 2000). The host shapes the bacterial population by providing nutrients to allow proliferation while affecting immune responses to limit population size and restrict areas of colonization (Silver and Graf, 2011; Nyholm and Graf, 2012; Graf, 2016). Bacteria modify the host’s response by inducing or limiting the expression of proteins, surface structures and signaling molecules. In leeches, different components of the innate immune system and ingested blood meal act in concert to limit microbe colonization and expansion (Indergand and Graf, 2000; Silver and Graf, 2011; Tasiemski et al., 2015).

Many innate immune system components likely to play a role in dominant symbiont selection have been identified in *Hirudo* spp. (Nyholm and Graf, 2012). In the crop, bacteria are phagocytosed by circulating hemocytes (**Figure 1B**). *A. veronii* expresses a type 3 secretion system (T3SS) that is critical for avoiding this phagocytosis (Silver et al., 2007a). In addition, a number of antimicrobial peptides have been identified in *H. medicinalis* and related hirudiniform leeches (Tasiemski et al., 2004, 2015; Schikorski et al., 2008) including salivary lectins (Min et al., 2010), theromyzin (TMZ), theromacin (TMC), allograft inflammatory factor-1 (AIF-1), neuromacin (NMC), lumbricin (LUMB). TMC and NMC are active against both Gram-positive and -negative bacteria through pore-forming and aggregate-forming mechanisms respectively (Jung et al., 2012). TMZ is active against Gram-positive bacteria while LUMB is active against a broad range of microorganisms (Cho et al., 1998). NMC, TMZ, and LUMB are all repressed in the leech crop when *A. veronii* is present (Tasiemski et al., 2015), suggesting that their expression may be partially regulated by this bacterium.

Putative lipopolysaccharide binding/bactericidal perme-ability increasing proteins (LBP/BPI) have been identified that may be involved in signaling or bacteriolysis. Four putative toll-like receptors (TLRs) have also been identified in the host transcriptome and may be important in recognizing microbe-associated molecular patterns (Schikorski et al., 2008; Macagno et al., 2010; Hibsh et al., 2015). A number of anti-human CD antibodies indicative of macrophages and natural killer cells also cross-react with leech hemocytes, suggesting the ability of self/non-self recognition (de Eguileor et al., 2000a,b).

In addition to leech-produced compounds, factors present in the ingested blood meal and those produced by symbionts help to shape the microbiome. The complement from ingested blood is active inside the leech gut and restricts the proliferation of certain non-symbiotic species and serum-sensitive *Aeromonas* mutants (Indergand and Graf, 2000; Braschler et al., 2003). A recent study by Tasiemski et al. (2015) suggested that antimicrobial peptides produced by *A. veronii* also contribute to restricting the species diversity of the leech microbiome. This observation confirms early findings by Büsing et al. (1953) who suggested that the leech digestive-tract symbionts prevent other bacteria from colonizing the leech digestive tract.

The combination of host-derived innate immune response, blood-meal-derived innate immune components, and symbiont-produced compounds suggests that a complicated network of factors controls the composition and density of symbionts. While the involvement of most factors remains to be verified, these data suggest that even in the absence of the canonical adaptive immune system there are many layers of antimicrobial compounds that a successful symbiont must overcome.

## Regulation

Bacteria continually monitor and respond to environmental cues in order to adapt to changing conditions. The ability of *A. veronii* isolates to beneficially associate with leeches and zebrafish (Graf, 1999; Roeselers et al., 2011), persist as free-living bacteria in freshwater aquatic environments, and exhibit virulence toward various vertebrates (Janda and Abbott, 2010) suggests the need for regulatory systems which detect different environments and regulate gene expression accordingly. Three predicted *A. veronii* Hm21 regulatory elements are implicated in colonization of the leech crop through mini-Tn*5* STM (**Table 1**) (Silver et al., 2007b).

An encoded RNase II disrupted in JG574 is a member of the RNase II/RNB-family of 3′-5′ exoribonucleases, which function in mRNA turnover (**Table 1**). Several enzymes from this group, particularly RNase R homologs, are implicated as virulence factors in various pathogens (Matos et al., 2014). Interestingly, an RNAse II from the nematode symbiont *Photorhabdus temperata* is needed for full insect virulence but not symbiosis (Hurst et al., 2015). While the specific role for RNase II in *A. veronii* symbiosis is unknown, it may act through a mechanistic pathway similar to that occurring in other bacterial pathogens.

Another factor with a somewhat ambiguous function is a YchF-GTPase homolog disrupted in JG697, a member of a group of highly conserved GTPases having unique substrate specificity for ATP over GTP (**Table 1**). YchF associates with ribosomes in *E. coli*, though the importance of this interaction is unclear (Verstraeten et al., 2011). Interestingly, the ATPase activity of this enzyme was recently shown to be redox regulated, and a wider role for YchF in inhibition of the oxidative stress response has been proposed (Wenk et al., 2012; Hannemann et al., 2016). Although the function and impact of the *A. veronii* Hm21 YchF homolog on leech colonization remains unexplored, since the mutant displayed similarly reduced CI values in both the leech and blood a general defect may be present (Silver et al., 2007b).

## Host-Symbiont Colonization/Virulence Factors

The ability of *A. veronii* to proliferate as a symbiont in digestive tracts of leeches and zebrafish (Bates et al., 2007; Roeselers et al., 2011; Nelson and Graf, 2012) and cause diseases in fish and mammals (Janda and Abbott, 2010; Beaz-Hidalgo and Figueras, 2013; Hossain et al., 2014) provides an opportunity to identify colonization factors for beneficial associations as well as pathogenic ones (Hentschel et al., 2000; Silver et al., 2007a). *A. veronii* Hm21, a strain isolated from *H. verbana*, displays virulence in multiple model systems including *Galleria mellonella* (wax-worms), intraperitoneal mouse injections, and an *in vitro* mammalian cell cytotoxicity model (Silver et al., 2007a, 2011). Unlike other pathogens that are highly host-specific, there is strong phylogenetic evidence for host-switching and virulence factor horizontal transmission between *Aeromonas* species (Silver et al., 2011; Martino et al., 2013). This high frequency of horizontal gene transfer makes ascribing clear ‘species-edge’ delineations very difficult when using a single or few housekeeping genes (Martino et al., 2013; Colston et al., 2014; Beaz-Hidalgo et al., 2015).

Several secretion systems have been identified as being necessary for virulence and symbiosis, including the T2SS and T3SS. The importance of the T2SS in colonization with regards to erythrocyte lysis was discussed earlier. Another STM mutant (JG752) was disrupted in a T3SS structural component and was unable to colonize the leech (**Table 1**). Unlike wild-type cells, JG752 was phagocytosed by leech hemocytes, indicating a specific role for the T3SS in *Aeromonas* evasion of the host immune system (Silver et al., 2007a). JG752 also exhibited decreased lysis of murine macrophage cells and decreased virulence in mice relative to the wild-type strain (Silver et al., 2007a), illustrating the dual importance of this factor in both symbiosis and pathogenesis. Identification and characterization of the complement of T3SS effectors will be crucial for understanding how *A. veronii* specifically utilizes this colonization/virulence factor to either promote persistence in the leech or opportunistic virulence in other organisms.

An additional secretion system, the T6SS, is an important factor used by bacteria to attack both prokaryotic and eukaryotic cells. New and diverse effector/immunity proteins for the T6SS are routinely being discovered (Cianfanelli et al., 2016). A re-evaluation of the sequence surrounding the disrupted locus of JG573 identified a rearrangement hotspot protein (Rhs) encoding gene located within a cluster of T6SS genes which include an encoded hemolysin co-regulated protein (Hcp), a valine-glycine repeat protein G (VgrG), and a PAAR-repeat protein (**Table 1**). These are all integral components of the T6SS delivery apparatus and can associate with T6SS effectors. Rhs proteins typically contain multiple domains and may possess a range of effector domains, which disrupt target cell processes (Koskiniemi et al., 2013). In *A. hydrophila*, the T6SS has been shown to be important in virulence (Suarez et al., 2010). The severe competition defect (>100-fold) displayed by the JG573 mutant indicates that the T6SS is an important *Aeromonas* colonization factor in the leech, though whether it is required for interaction with other bacterial cells or those of the host immune system remains to be seen.

## Leech Therapy and *Aeromonas* Infections

Leech use as a medical practice dates back to ancient Egypt (Whitaker et al., 2004b). Since then, blood-letting has transformed from a religious experience to rid the body of disease and ‘ill humors’ to the contemporary practice of hirudotherapy. *H. medicinalis* leeches gained FDA-approval in 2004 and today leeches are widely used in US hospitals for treatment of compromised vasculature. Leeches are especially used after free-tissue transfer such as replanted digits, ears, facial and breast tissue (Whitaker et al., 2004a,b, 2012; Nelson and Graf, 2012). Leech therapy provides great therapeutic benefits during post-operative remediation, with studies demonstrating an associated decrease in the rate of graft failures and risk of amputation (Whitaker et al., 2011, 2012). Leeches are applied to the venous-congested sites and bite the tissue to withdraw obstructive blood while simultaneously secreting an anticoagulating agent and vasodilators to further reduce circulatory obstruction and facilitate blood flow through the area (Michalsen et al., 2008; Whitaker et al., 2011, 2012). One survey analyzed 277 case reports to quantify the efficacy of leech therapy and found that 78% of cases resulted in success, where transferred tissue was salvaged and no complications occurred (Whitaker et al., 2012). However, the advantages of leech therapy are confounded by more recent and widely reported occurrences of leech-borne infections at the bite wound, which may cause septicemia in the patient when left untreated.

Complications of leech therapy occur in part due to bacterial infections, which are thought to originate from the microbial community of the *H. verbana* crop. The incidence of infections in the literature ranges from 2 to 36% of cases (Whitaker et al., 2011). Prophylactic antibiotics reduce incidence of infections to the lower end of this range, though in some clinical settings no prophylactics are used at all (Whitaker et al., 2011). The occurrence of infections dramatically reduces the ability to salvage new tissue and thus jeopardizes the successful outcome of the surgery (Whitaker et al., 2011). Even with prophylactic antibiotic use, recent case reports describe severe infection of the tissue graft, which in some cases resulted in amputation of the limb or digit (**Table 2**).

**Table 2:** Recently published case reports of Ciprofloxacin^R^ *Aeromonas* spp. cultured in association with medicinal leech therapy.

Reference	No. of case reports	Patient case conditions	Prophylaxis used	Treatment that cleared infection	Isolate(s) cultured	Geographic location	Year published
Wang et al., 2011	1	Mandibulectomy with planned tissue flap reconstruction, infection and necrosis of the flap	Ciprofloxacin	Cefepime	*A. hydrophila*	Missouri, USA	2011
Sartor et al., 2013	1	Infection of skin flap of hand crush injury	Ciprofloxacin	Cotrimoxazole	*A. hydrophila*	Marseille, France	2013
Giltner et al., 2013	1	Mandibular osteotomy, necrosis of mandibular flap and wound surrounding the distraction arm device	Ciprofloxacin	Vancomycin	*A. hydrophila, Morganella morganii*	California, USA	2013
Wilmer et al., 2013	1	Amputation of three digits and necrosis of amputation sites	Ciprofloxacin	Co-trimoxazole	*A. hydrophila*	British Columbia, Canada	2013
Patel et al., 2013	1	Breast reconstruction, infection of the implant	Ciprofloxacin, vancomycin	Aztreonam	*A. hydrophila*	Washington D.C., USA	2013
van Alphen et al., 2014	2	Replantation of four fingers resulting in flap necrosis after leech therapy, followed by amputation	Ertapenem	Ceftriaxone and co-trimoxazole	*A. hydrophila*	Minnesota, USA	2014
		Replantation of two fingers failed following leech therapy, amputation	Ciprofloxacin	Cefepime, metronidazole, vancomycin; followed by ceftriaxone	*A. hydrophila, Proteus vulgaris, Morganella morganii*		


## Clinical Reports of *Aeromonas* Infections

The most commonly isolated bacteria from infected leech bite wounds belong to the *Aeromonas* genus, including the fish and human pathogen *A. hydrophila* (Whitaker et al., 2012). *A. hydrophila* was reported in 88% of case reports involving infections, followed by *A. veronii* and *A. sobria* (Whitaker et al., 2012). These numbers may be affected by species-misidentifications resulting from inadequate characterization methods of the strains within this genus (Silver et al., 2011; Colston et al., 2014). Surprisingly, a recent *Aeromonas* infection following a pharyngectomy was reported to cause pneumonia in addition to tissue flap infection (Van Derick and Dasgupta, 2016). The risks associated with leech-borne *Aeromonas* infections have led many hospitals to adopt the use of ciprofloxacin (Cp), for prophylactic treatment as a standard practice before leech application. Cp is a widely used broad-spectrum fluoroquinolone and has been shown to inhibit *Aeromonas*, making it very useful in leech therapy prophylaxis (Whitaker et al., 2011). Until the 2000s, *Aeromonas* resistance to Cp was largely unreported, and to our knowledge, no Cp^R^ (Cp resistance) cases associated with leech therapy were published until 2011.

From 2011 to 2016, infections by Cp^R^ *A. hydrophila* were reported in eight patients following leech therapy where Cp was used as prophylaxis in the United States, Canada, and France (Wang et al., 2011; Giltner et al., 2013; Patel et al., 2013; Sartor et al., 2013; Wilmer et al., 2013; van Alphen et al., 2014), contributing to concerns of a rise in antibiotic resistant-*Aeromonas* infections (**Table 2**). These infections occurred at a range of body sites and to date have been successfully controlled by administering either individual antibiotics or combinations (see **Table 2** for details). However, serious consequences of Cp^R^ *Aeromonas* infections following leech therapy can occur, including complete graft necrosis and amputation. For example, in 2013 a patient receiving leech therapy after mandibular surgery acquired an infection that resulted in tissue necrosis and required immediate treatment with more effective antibiotics (Van Derick and Dasgupta, 2016). In light of these nosocomial infections caused by *Aeromonas* strains, some research has been done to determine the genetic factors underlying an increase in Cp^R^. Several studies suggested the importance of point mutations in *gyrA* and *parC* as well as the acquisition of plasmid encoded resistance genes such as *qnrS* in Cp^R^ (Giraud et al., 2004; Arias et al., 2010). Sartor et al. (2013) hypothesize a rise in resistance could originate from exposure to fluoroquinolones present in the blood of poultry used to feed leeches at the raising facility. However, further work needs to be performed to directly link clinical isolates to leeches and the genetic basis of the Cp^R^ in these strains remains to be determined.

## Conclusion

A microbiome that is consistently dominated by two species, access to the genome sequences, culturability of dominant symbionts, and an ability to genetically manipulate *Aeromonas* are reasons for which the medicinal leech is an excellent model for studying the microbe–host interactions in digestive-tract symbioses. Owing in part to ease of culturing and genetic manipulation of *Aeromonas*, the nutrition, colonization, and persistence factor requirements of this symbiont are much better understood than others, such as *M. hirudinis*.

Global interrogative methods such as metagenomic and metatranscriptomic analyses have proven invaluable in identifying host and symbiont responses relating to altered gut microbiome composition and physiology. Future elucidation of more complex interactions and interrelations amongst symbionts and the leech host, such as nutrient metabolic cascades and specific immune responses, will require increased application of biochemical, molecular and genetic tools. We now have a substantial understanding and appreciation of the diversity of the leech microbiome. Future research should aim to identify parameters that contribute to the establishment of the leech gut microbiome.

Lastly, despite the proven medical benefits of leech therapy, recognition of the leech as a vector for wound infections following reconstructive surgery has led to a greater appreciation for a need to proactively minimize this undesired outcome. To this end, prophylactic administration of ciprofloxacin is common practice in leech therapy. However, since we now know medicinal leech associated Aeromonad fluoroquinolone resistance is on the rise, medical practices will need to be modified to prevent avoidable infections.

## Author Contributions

All authors listed have made substantial, direct and intellectual contribution to the work, and approved it for publication.

## Conflict of Interest Statement

The authors declare that the research was conducted in the absence of any commercial or financial relationships that could be construed as a potential conflict of interest.
